# ^11^C-choline *vs*. ^18^F-FDG PET/CT in assessing bone involvement in patients with multiple myeloma

**DOI:** 10.1186/1477-7819-5-68

**Published:** 2007-06-20

**Authors:** Cristina Nanni, Elena Zamagni, Michele Cavo, Domenico Rubello, Paola Tacchetti, Cinzia Pettinato, Mohsen Farsad, Paolo Castellucci, Valentina Ambrosini, Gian Carlo Montini, Adil Al-Nahhas, Roberto Franchi, Stefano Fanti

**Affiliations:** 1 Nucler Medicine, PET Unit, Policlinico S. Orsola-Malpighi, Bologna University, Italy; 2 Haematology and Oncology "Seràgnoli Institute", Policlinico S. Orsola-Malpighi, Bologna University, Italy; 3 Nuclear Medicine, PET Unit, S. Maria della Misericordia Rovigo Hospital, Istituto Oncologico Veneto (IOV)-*IRCCS*, Italy; 4 Department of Nuclear Medicine, Hammersmith Hospital, London, UK

## Abstract

**Background:**

Multiple Myeloma (MM) is a B cell neoplasm causing lytic or osteopenic bone abnormalities. Whole body skeletal survey (WBSS), Magnetic resonance (MR) and ^18^F-FDG PET/CT are imaging techniques routinely used for the evaluation of bone involvement in MM patients.

**Aim:**

As MM bone lesions may present low ^18^F-FDG uptake; the aim of this study was to assess the possible added value and limitations of ^11^C-Choline to that of ^18^F-FDG PET/CT in patients affected with MM.

**Methods:**

Ten patients affected with MM underwent a standard ^11^C-Choline PET/CT and an ^18^F-FDG PET/CT within one week. The results of the two scans were compared in terms of number, sites and SUV_max _of lesions.

**Results:**

Four patients (40%) had a negative concordant ^11^C-Choline and ^18^F-FDG PET/CT scans. Two patients (20%) had a positive ^11^C-Choline and ^18^F-FDG PET/CT scans that identified the same number and sites of bone lesions. The remaining four patients (40%) had a positive ^11^C-Choline and ^18^F-FDG PET/CT scan, but the two exams identified different number of lesions. Choline showed a mean SUV_max _of 5 while FDG showed a mean SUV_max _of 3.8 (P = 0.042). Overall, ^11^C-Choline PET/CT scans detected 37 bone lesions and ^18^F-FDG PET/CT scans detected 22 bone lesions but the difference was not significant (P = 0.8).

**Conclusion:**

According to these preliminary data, ^11^C-Choline PET/CT appears to be more sensitive than ^18^F-FDG PET/CT for the detection of bony myelomatous lesions. If these data are confirmed in larger series of patients, ^11^C-Choline may be considered a more appropriate functional imaging in association with MRI for MM bone staging.

## Background

Multiple myeloma (MM) is a B cell neoplasm involving bones in more than 80% of cases. Patients frequently present with a single or multiple lytic bone lesions causing bone pain, pathological fractures and hypercalcaemia [1-5]. Bone abnormalities (lytic or osteopenic) are one of the myeloma related organ dysfunction [6] and are responsible for low quality of life due to severe pain and high incidence of fractures, and this is particularly dangerous if located in the spine. The incidence of vertebral fractures can be reduced with bisphosphonates that are now available in the therapeutic armamentarium of MM.

Bone lesions are usually evaluated with a spectrum of imaging techniques, among which whole body skeletal survey (WBSS) and spine and pelvis Magnetic Resonance Imaging (MRI) are the most widely used. [7].

WBSS is known to be relatively insensitive for bone damage detection as only those lesions characterized by a high re-absorption rate (therefore appearing at a late stage) are visible. Furthermore, WBSS, being a planar technique, can easily underestimate bone involvement especially within the spine where overlying tissues and rib cage hinder the assessment of osteolysis. In addition, WBSS cannot distinguish between idiopathic osteoporotic vertebral fractures and fractures due to MM and is not suitable to assess the response to therapy.

Spine MRI, which was recently integrated in the Durie and Salmon PLUS staging system, is proved to have a very good sensitivity compared to WBSS especially at disease onset [8-13]. The main limitations of MRI are the inability to perform the scan in the presence of metallic prosthesis or in case of severe claustrophobia. More importantly, MRI is limited by the partial field of view that includes only the spine and the pelvis. The skull, femura, humeri, clavicles and ribs are often affected by lytic lesions but are not included in MRI field of view. Whole Body MRI is now available for a complete skeletal survey, but is rarely employed on a routine basis.

Nuclear medicine imaging techniques were also used to assess MM bone involvement. ^99m^Tc-diphosphonate bone scan and ^67^Ga-citrate scan were found to be unreliable due to minimal osteoblastic activity and hypovascularity of lesions. ^99m^Tc-Sestamibi whole-body scan is more accurate but the low spatial resolution limits the identification of small lesions. Furthermore, the image interpretation can be difficult due to the low tracer uptake within the lesions and to the high physiological liver uptake that can mask vertebral and right rib lesions. Therefore, nuclear medicine tests have not gained widespread acceptance [14-20].

In recent years, ^18^F-FDG PET and PET/CT were used as possible novel strategy for MM evaluation. ^18^F-FDG PET is a total body imaging technique that can detect both medullary and extra-medullary lesions and has been found useful for improving staging accuracy. Durie *et al*. in 2002 demonstrated that a negative ^18^F-FDG PET scan predicts stable monoclonal gammopathy of indeterminate significance (MGIS), identifies small lesions not detected by WBSS, identifies extra-medullary lesions related to poor prognosis and predicts an early relapse if it was positive after therapy [21]. These results were confirmed by other recent publications [22,23].

As stated before, ^18^F-FDG PET/CT is useful to correctly stage MM with increased accuracy of bone lesion detection at disease onset. It is more sensitive than WBSS and includes all the bones located out of the MRI field of view [24].

Despite its sensitivity, the uptake of FDG assessed with the maximum Standardized Uptake Value (SUV_max_) can be very low, sometimes even comparable to the SUV_max _of a benign lesion. Distinguishing between a benign lesion and a low-metabolic MM lesion can therefore be difficult to achieve.

^11^C-Choline is a radiolabelled PET tracer compound that is clinically used for the evaluation of relapse of prostate cancer. As with MM, prostate cancer does not show a significant increase of ^18^F-FDG uptake, but is characterized by a high ^11^C-Choline uptake [25]. Interestingly, a recently published case report has shown increased ^11^C-Choline uptake in a solitary plasmacytoma of bones [26].

The aim of our study was to assess the possible added value and limitations of ^11^C-Choline compared with ^18^F-FDG PET/CT in patients affected by MM.

## Patients and methods

Between November 2004 and June 2006, we studied 10 patients (7 males and 3 females, mean age 58 years) affected with MM. They underwent ^11^C-Choline PET/CT and ^18^F-FDG PET/CT within one week (in most cases on the same day). Four of the patients were evaluated at completion of initial therapy, 2 during follow-up and 4 at disease relapse. At disease onset, all the patients were in Durie and Salmon stage III due to the presence of bone lesions. For the 11C-Choline scan, all patients provided informed consent for participation and anonymous publication of data.

Patients were injected with 5.3 MBq/Kg ^11^C-Choline iv and scanned after an uptake period of 5 minutes. Data acquisition was performed with a dedicated PET/CT tomograph (GE, Discovery). Images were acquired in 2D mode for 4 min per bed position, and attenuation correction was performed with a CT-based method (120 kV, 80 mA). Each PET/CT scan was read by two nuclear medicine physicians and the reports agreed upon by consensus. Each visible area of focal ^11^C-Choline uptake in bone (excluding joints) was considered positive for a myelomatous lesion. The SUV_max _was calculated using the following formula:

### Tissue concentration (MBq/g)/injected dose (MBq)/body weight (g)

At least 4 hours after the ^11^C-Choline scan, the patients were injected with 5.3 MBq/Kg ^18^F-FDG iv. None of the patients was diabetic and the fasting time required for ^11^F-FDG studies was at least 4 hours. The uptake time was 60–90 minutes and the data acquisition was performed as for the ^11^C-Choline scan.

^11^C-Choline scan results were compared to ^18^F-FDG scan results in terms of number of lesions and SUV_max_. The SUV_max _cut-off was 1.0 for ^11^C-Choline studies (the higher uptake that we measured in normal bones) while all the areas of focal uptake were interpreted as positive for myeloma in ^18^F-FDG scan unless they were at sites of known accumulation. The latter include the kidneys and bladder, gastrointestinal tract, and skeletal areas showing symmetric joint uptake, especially within the shoulder girdle [21]. A mild diffuse increase in bone marrow activity was not interpreted as positive for myeloma as it is a frequent finding even in normal patients [27].

The CT attenuation correction map was not used as a reference diagnostic tool. Several bone lesions are normally detected by PET at an early stage before these ere detectable with morphological imaging such as CT since density alteration occurs much later than metabolic activity. Furthermore, patients evaluated after being treated with a specific therapy may present with persistent osteolytic lesions on CT that do not show significant metabolic activity any more.

All patients had at least one-year follow-up and underwent several imaging procedures according to the clinical decision and needs.

### Statistical analysis

Statistical significance of differences in ^11^C-Choline SUV_max _and ^18^F-FDG SUV_max _was determined using the Student *t *Test. A two-tailed Mann-Whitney test was used to compare the number of lesions detected with ^11^C-Choline and ^18^F-FDG. The minimal level of significance was a *P *< 0.05.

## Results

The mean number of lesions detected per patient in the entire group was 3.7 for ^11^C-Choline and 2.2 for ^18^F-FDG (P = 0.8). Considering only positive patients, the mean number of lesions detected per patient was 7.4 for ^11^C-Choline and 3.7 for ^18^F-FDG (Table 1).

**Table 1:** Number of bone lesions detected by ^11^C-Choline PET/CT and ^18^F-FDG PET/CT patient by patient.

**Patient**	**^11^C-Choline PET/CT**	**^18^F-FDG PET/CT**
1	0	0
2	8	1
3	0	0
4	2	1
5	0	0
6	11	11
7	10	2
8	0	0
9	6	6
10	0	1
**Total**	**37**	**22**

In 4/10 patients (40%) there was a negative concordant ^11^C-Choline and ^18^F-FDG PET/CT scans. These findings were consistent with clinical, laboratory and radiological data indicating a complete remission at the time of imaging. Of those four patients, three were evaluated after therapy and one during follow-up.

In 2/10 patients (20%), evaluation was performed due to suspicion of disease relapse and both ^11^C-Choline and ^11^F-FDG PET/CT scans were positive. In this group, both techniques identified the same number and sites of bone lesions.

The remaining 4/10 (40%) patients had a positive ^11^C-Choline and ^18^F-FDG PET/CT scans, but the two techniques identified a different number of lesions. In 3/4 patients, ^11^C-Choline identified more lesions compared to ^18^F-FDG (8 vs. 1; 2 vs. 1; 10 vs. 2), while in 1/4 patient ^18^F-FDG detected a disease relapse within the pelvis that was negative with ^11^C-Choline. Of these four patients, 2/4 were evaluated due to suspicion of disease relapse, 1/4 following therapy and 1/4 during follow-up.

Table 2 shows the SUV max on a lesion by lesion basis for 11C-Choline scans and 18F-FDG scans (Table 2). ^11^C-Choline showed a mean SUV_max _of 5, while ^18^F-FDG showed a mean SUV_max _of 3.8 and the difference was statistically significant (p = .042). The SUV_max _of visually detectable lesions ranged from 1.1 to 19.2 for ^11^C-Choline and from 2 to 13.7 for ^18^F-FDG. (Table 2).

**Table 2:** Sites of lesions and SUV_max _(^11^C-Choline and ^18^F-FDG) on a lesion by lesion basis. Bold: SUV_max _of positive lesions. Non Bold: SUV_max _of negative areas.

**Patient number**	**Gender**	**Age (years)**	**Disease stage**	**Therapy**	**Indication to PET**	**Follow-up (months)**	**Confirmation of lesions**	**Number of lesions**	**Site of lesions**	**SUVmax Choline PET**	**SUVmax FDG PET**
**1**	Male	61	IgA/lamda IIIA	Chemotherapy + double autotranspalnt	Post-therapy	19	Clinical follow-up	0			
**2**	Male	55	IgA/lamba IIIA	Chemotherapy + autotranspalnt	Suspect relapse	20	Clinical follow-up	1 2 3 4 5 6 7 8	pleura soft tissues humerus humeral head soft tissues Ribs D11 D12	7.0 4.2 5.1 6.0 3.0 4.7 2.5 2.7	1.0 2.0 1.3 1.9 0.8 1.5 1.0 1.0
**3**	Male	56	IgA/lamba IIIA	Chemotherapy + autotranspalnt	Post-therapy	8	Whole body X-rays		0		
**4**	Male	72	Solitary plasmacytoma of bones	Radiotherapy	Follow-up	31	Magnetic resonance imaging	9 10	sacrum D8–D10	5.6 2.0	2.8 1.0
**5**	Male	62	IgG/K IA	Chemotherapy + autotranspalnt	Follow-up	16	Clinical follow-up	0			
**6**	Female	55	IgG/lamba IIIA	Chemo-radiotherapy	Suspect relapse	16	Clinical follow-up	11 12 13 14 15 16 17 18 19 20 21	skull scapula clavicle humerus soft tissues soft tissues ribs sternum pelvis sacrum femur	1.3 4.2 2.8 3.6 5.5 4.5 6.5 4.0 7.5 6.0 5.7	3.5 9.7 6.6 4.5 13.7 12.3 6.5 3.0 4.9 5.8 3.1
**7**	Female	57	IgG/lamba IIIA	Chemotherapy + autotranspalnt	Suspect relapse	16	FDG PET/CT	22 23 24 25 26 27 28 29 30 31	scapula sternum clavicle ribs D4–D8 L4 L5 pelvis sacrum femur	1.3 1.8 2.4 3.0 2.2 2.3 2.2 2.9 1.1 3.0	1.0 3.4 1.2 2.5 1.0 1.7 1.9 0.9 1.6 1.7
**8**	Male	59	IgG/lamba IA	Chemotherapy	Post-therapy	8	FDG PET/CT	0			
**9**	Female	49	IgA/K IIIA	Chemotherapy + autotranspalnt	Suspect relapse	8	FDG PET/CT	32 33 34 35 36 37	skull clavicle scapula ribs pelvis femur	15.0 12.9 7.5 12.4 4.6 19.2	5.8 9.1 2.5 8.5 3.0 7.6
**10**	Male	53	Solitary plasmacytoma of bones	Radiotherapy	Post-therapy	1	Magnetic resonance imaging	38	sacrum	0.9	4.9
									**Mean**	**5.0**	**3.8**
									***p***	**0.042**	

Overall, ^11^C-Choline PET/CT scans detected 37 bone lesions while ^18^F-FDG PET/CT scans detected 22 bone lesions. This difference, however, was not statistically significant and the *P *value was 0.8.

All the patients underwent a follow-up (1 month to 1 year long) by repeating ^18^F-FDG PET/CT, MRI or CT. No false positive findings were observed for the ^18^F-FDG or ^11^C-Choline.

## Discussion

Our preliminary results show that ^11^C-Choline PET/CT detected more myelomatous lesions than ^18^F-FDG PET/CT in our group of 10 patients. Although the difference between the two tracers was not statistically significant in terms of mean number of lesions detected, it is interesting to note that ^11^C-Choline detected more lesions than ^18^F-FDG in patient 2 and 7 (8 *vs*. 1 and 10 *vs*. 2), radically changing these patients' management.

**Figure 1:**
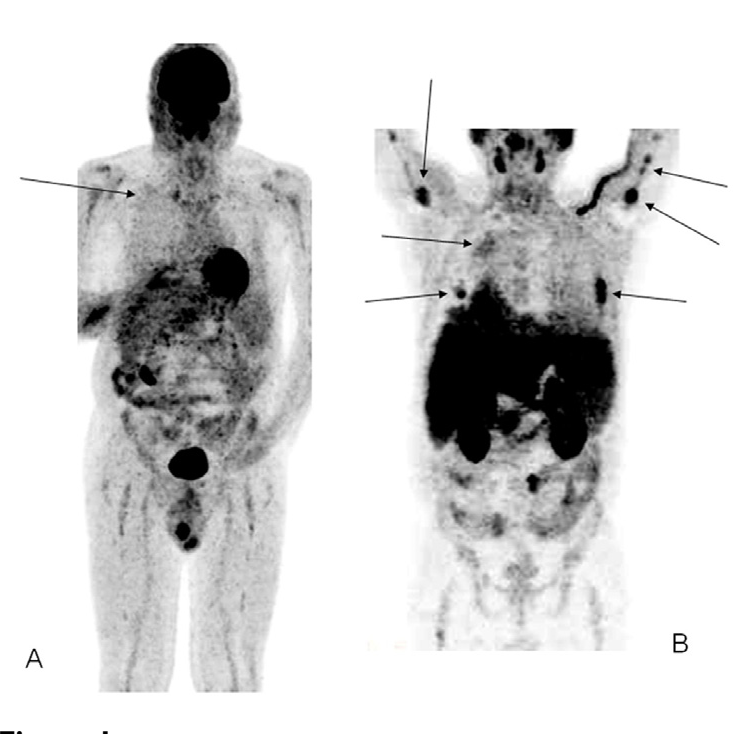
^18^F-FDG PET scan (A) and ^11^C-Choline PET scan (B) of a MM patient. A) a small area of minimal uptake is detectable in dorsal soft tissues (black arrow), B) several areas of increased uptake are detected in bones, soft tissues and pleura (black arrows).

One patient turned out positive for several pleural lesions, soft tissue involvement and bone lesions on ^11^C-Choline, while ^18^F-FDG detected only the soft tissue lesion (Figure 1). Another patient turned out positive for several bone lesions on ^11^C-Choline scan, while ^18^F-FDG detected only a rib and a sternal lesion. On average, SUV_max _was significantly higher for ^11^C-Choline-positive lesions compared to ^11^F-FDG-positive lesions (5.0 vs. 3.8), and this is an unusual finding as ^11^C-Choline and 18F-FDG-positive lesions behave in different ways.

It is not clear why myelomatous lesions demonstrate such high ^11^C-Choline uptake compared to ^18^F-FDG. Sasagawa *et al*, in a series of 16 patients affected by MM, demonstrated that the serum levels of lysophospholipids were significantly increased compared to normal patients [28]. Recently, Hideshima *et al*. showed that perifosine, an alkylphospholipid, is active in-vitro against myelomatous cells by inhibiting the phosphatidilinositol 3-kinase/Akt, a mitogen-activated protein kinase which mediates MM cell resistance to conventional therapies [29].

These data suggest that phospholipids are strongly involved in the metabolism of myelomatous cells, especially in the modulation of intracellular growth signal transduction pathways.

Choline is a small molecule precursor of phospholipids and its uptake is increased in proliferating cells because it is involved in membrane metabolism and growth (increased during the mitotic process) that is significantly altered in MM lesions.

The additional value of sensitive bone imaging techniques in patients affected with MM is still not well defined, but remains part of the routine assessment of disease activity. However, recent studies suggest that the number of bone lesions is related to the prognosis and that the functional measurement of reduction in metabolism is a long term predictive parameter of therapy response [30].

If this concept is confirmed in studies with larger number of patients, the role of a sensitive technique that assesses the whole body, such as ^11^C-Choline, could acquire importance in patients affected by MM. In particular, it may help to customise an early aggressive therapy in case of multiple bone lesions to prevent a disease relapse or loss of bone mineral density resulting in multiple fractures.

The main disadvantage of ^11^C-Choline is the physiological liver uptake that prevents detection of hepatic lesions that may occur, though rarely, in MM patients. Furthermore, the role of ^11^C-Choline PET for the detection of infiltrative pattern of the spine, not characterized by distinct focal lesions, needs further assessment as our small series did not include any patient with such pattern on MRI. This may prove to be useful as ^18^F-FDG PET is not sensitive in the identification of infiltrative pattern of the spine.

**Figure 2:**
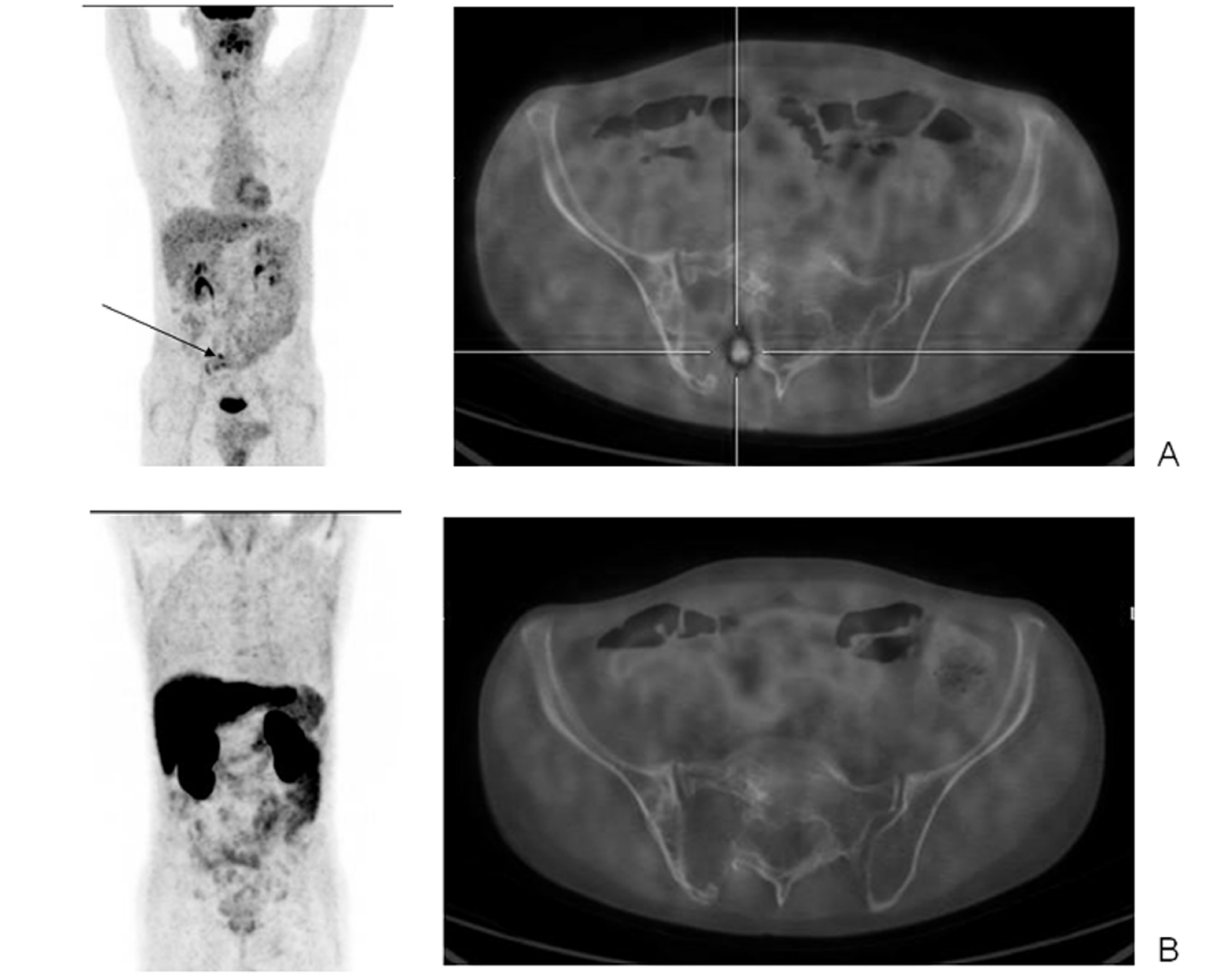
^18^F-FDG PET scan (A) a small area of minimal uptake is detectable in the pelvis (black arrow) B) ^11^C-Choline PET scan of a MM patient. no Choline uptake is shown.

One patient had a positive ^18^F-FDG PET scan showing a focal area of increased uptake located in the pelvis and a negative ^11^C-Choline PET scan (Figure 2). The significance of this mismatch is difficult to assess due to the short period of follow up. It has been suggested that a lesion with an initially negative ^18^F-FDG scan that shows uptake at a later stage may have developed de-differentiation of cancer cells and this may explain this discordant finding. The prognosis in such cases is inversely correlated with the SUV_max _[31,32].

## Conclusion

According to our preliminary data, ^11^C-Choline PET/CT appears to be more sensitive than ^18^F-FDG PET/CT for the detection of bone myelomatous lesions. If these data can be confirmed in a larger series of patients, ^11^C-Choline could be the most appropriate functional imaging in combination with MRI for MM bone staging.

## Competing interests

The author(s) declare that they have no competing interests.

## Authors' contributions

**EZ, MC, PT **managed the patients and performed clinical and biochemical examinations. **CN, RF, VA **participated in the design of the study. **CP **performed the quality controls of the PET/CT system. **MF, PC, GCM **performed the PET/CT examinations and drafted the manuscript. **SF, DR, AA **participated to the coordination of the study and helped to draft the study. All authors read and approved the final manuscript.
